# Co-existence of PrP^D^ types 1 and 2 in sporadic Creutzfeldt-Jakob disease of the VV subgroup: phenotypic and prion protein characteristics

**DOI:** 10.1038/s41598-020-58446-0

**Published:** 2020-01-30

**Authors:** Ignazio Cali, Gianfranco Puoti, Jason Smucny, Paul Michael Curtiss, Laura Cracco, Tetsuyuki Kitamoto, Rossana Occhipinti, Mark Lloyd Cohen, Brian Stephen Appleby, Pierluigi Gambetti

**Affiliations:** 1 Department of Pathology, Case Western Reserve University, School of Medicine, Cleveland, OH 44106 USA; 2 Department of Neurology, Case Western Reserve University, School of Medicine, Cleveland, OH 44106 USA; 3 Department of Psychiatry, Case Western Reserve University, School of Medicine, Cleveland, OH 44106 USA; 4 Department of Physiology and Biophysics, Case Western Reserve University, School of Medicine, Cleveland, OH 44106 USA; 5 National Prion Disease Pathology Surveillance Center, Case Western Reserve University, School of Medicine, Cleveland, OH 44106 USA; 6 Department of Advanced Medical and Surgical Sciences, University of Campania “L. Vanvitelli”, Caserta, 81100 Italy; 7 Department of Psychiatry, University of California, Davis, CA 95616 USA; 8 New York University, School of Medicine, New York, NY 10016 USA; 9 Department of Neurological Science, Tohoku University Graduate School of Medicine, Sendai, 980-8576 Japan

**Keywords:** Prions, Prion diseases

## Abstract

We report a detailed study of a cohort of sporadic Creutzfeldt-Jakob disease (sCJD) VV1–2 type-mixed cases (valine homozygosity at codon 129 of the prion protein, PrP, gene harboring disease-related PrP, PrP^D^, types 1 and 2). Overall, sCJDVV1–2 subjects showed mixed clinical and histopathological features, which often correlated with the relative amounts of the corresponding PrP^D^ type. However, type-specific phenotypic characteristics were only detected when the amount of the corresponding PrP^D^ type exceeded 20–25%. Overall, original features of types 1 (T1) and 2 (T2) in sCJDVV1 and -VV2, including rostrocaudal relative distribution and conformational indicators, were maintained in sCJDVV1–2 except for one of the two components of T1 identified by electrophoretic mobility as T1^21^. The T1^21^ conformational characteristics shifted in the presence of T2, inferring a conformational effect of PrP^D^ T2 on T1^21^. The prevalence of sCJDVV1–2 was 23% or 57% of all sCJDVV cases, depending on whether standard or highly sensitive type-detecting procedures were adopted. This study, together with previous data from sCJDMM1–2 (methionine homozygosity at PrP gene codon 129) establishes the type-mixed sCJD variants as an important component of sCJD, which cannot be identified with current non-tissue based diagnostic tests of prion disease.

## Introduction

The heterogeneity that characterizes sporadic Creutzfeldt-Jakob disease (sCJD), a group of diseases that accounts for 85–90% of all human prion diseases, is due to the existence of at least five clinically and pathologically distinct subtypes. According to a widely used molecular classification, each sCJD subtype is identified by the pairing of the patient’s genotype — MM, MV or VV — at the methionine (M)/valine (V) polymorphic codon 129 of the prion protein (PrP), with the type, 1 or 2, of the abnormal, disease-related PrP (PrP^D^). The 1 and 2 typing identifies the two basic PrP^D^ isoforms or strains associated with sCJD and most other prion diseases. The sCJD heterogeneity is further complicated by the frequent occurrence of type “mixed” cases where the disease phenotypes and PrP^D^ type 1 (T1) and 2 (T2) associated with two subtypes coexist in variable ratios in the same patient. Therefore, besides the five “pure” subtypes (i.e., -MM(MV)1, -VV1, -MM2, -MV2 and -VV2), sCJD includes the four mixed subtypes MM1–2, MV1–2 and VV1–2, as well as the MV2K-C (Table S1).

The existence of 1–2 (also designated 1 + 2) mixed subtypes of sCJD was established in the first comprehensive report on molecular subtyping of sCJD^1^ as well as by Puoti and coworkers^2^ (Table S1). In a subsequent study using type-specific antibodies, it was asserted that the coexistence of two types of PrP^D^ was a regular feature of CJD^3^. However, questions were raised as to whether the proteinase K (PK)-treatment used by Polymenidou *et al*. was adequate to completely hydrolyze the N-terminus of the PrP^D^ fragments with low or intermediate PK-resistance, which may have resulted in an overestimation of the prevalence of mixed cases^4–6^. In a subsequent study, we systematically examined prevalence and other features of the sCJDMM1–2 mixed cases under conditions that assured complete hydrolysis of all intermediate fragments with preservation of the protease-resistant core^4^. Our study showed that although the detection of PrP^D^ 1–2 mixed type is directly related to the number of brain regions analyzed, examination of at least six major brain structures—including cerebral cortex, neostriatum, thalamus and cerebellum—confirmed the existence of sCJDMM1 and -MM2 cases in which only PrP^D^ T1 and T2, respectively, were detected. Even so, nearly 40% of all sCJDMM1 and -MM2 cases combined were sCJDMM1–2, with PrP^D^ T1 and T2 found together in the same brain region or separate in different regions^4^. Overall, clinical and histopathological features were a mixture of those associated with sCJDMM1 and -MM2. However, detailed analyses indicated that type-related features such as disease duration and histopathological characteristics were directly correlated with the relative amounts of PrP^D^ T1 and T2. Furthermore, mixed cases where 25% or less of PrP^D^ T1 or T2 was found in the cerebrum demonstrated almost exclusively with the histopathological phenotype characteristic of the dominant PrP^D^ type. These findings were further corroborated by Parchi *et al*.^6^.

The study of PrP^D^ type-mixed cases is also relevant to prognosis and clinical management since it provides information on duration and prevailing symptomatology of these non-rare variants of sCJD. Furthermore, current evidence suggests that successful therapeutics for prion diseases not only need a reliable and early diagnosis that can distinguish individual sCJD subtypes but might also require to be tailored to the individual prion strains^7–9^. Therefore, considering their prevalence, mixed cases pose a challenge to early diagnosis and, possibly, treatment of prion diseases since currently no diagnostic test, short of tissue examination, can identify these cases^7–9^. Mostly from this perspective, we have leveraged a large sCJDVV cohort, the second most common sCJD genotype, to carry out a detailed study of sCJDVV1–2 mixed variant. This study revealed intriguing similarities and important differences between sCJDVV1–2 and sCJDMM1–2.

## Results

### Validation of sCJDVV1–2, -VV1 and -VV2 case cohorts

We selected a total of 31 cases comprising sCJDVV1, -VV1–2 and -VV2 from a sCJDVV cohort provided by the National Prion Disease Pathology Surveillance Center (NPDPSC). We established relative amount, distribution and physicochemical characteristics of the PK-resistant PrP^D^ (resPrP^D^) types 1 and 2 as well as the histopathological and clinical phenotypes in each case of the cohort. Data obtained from the -VV1–2 subtype were then compared with corresponding data from -VV1 and -VV2 subtypes (Tables 1 and S2). Over 90% of the 12 brain tissue samples required by the experimental design were available for examination in each of the three sCJDVV subtypes. Preliminary studies established that Abs 3F4 and 1E4 were suitable for quantifying resPrP^D^ types 1 and 2 (thereafter referred to as T1 and T2), respectively; however, depending on the analysis, they were supplemented with the antibodies (Abs) 12B2 and Tohoku-2 (To-2) that are specific to T1 and T2, respectively. Ancillary analyses were performed to definitively exclude the possibility that the -VV1–2 cohort comprised misclassified cases resulting from incomplete digestion of PrP^D^ (Supplementary Figure S1)^4,5^. Following these analyses, 18 cases were diagnosed with sCJDVV1–2, 5 with -VV1 and 8 with -VV2. All -VV2 and -VV1 selected cases harbored 100% resPrP^D^ T2 and T1 respectively, in all brain regions examined, while in -VV1–2 both T1 and T2 were detected in at least one of the three major brain regions selected (Table 1).

**Table 1:** Demographic data and percentage of resPrP^D^ T2 detected in the brain of sCJDVV1, sCJDVV2 and sCJDVV1-2.

sCJD subtype	Case #	Disease onset^a^	Disease duration^a^	% resPrP^D^ T2^b^			
				Cerebral cortex^a,c^	Subcortical regions^a,d^	Cerebellum^a^	Whole brain^a,e^
**VV2**	1-8	68 ± 8^f^	6 ± 2^f^	100	100	100	100
**VV1-2**	1	55	4	97	100	100	98
	2	70	3	100	100	76	98
	3	70	17	94	97	100	96
	4	66	4	93	99	100	95
	5	74	5	90	99	100	93
	6	73	7	100	62	100	93
	7	44	7	88	99	99	92
	8	45	5	87	100	100	91
	9	57	8	69	93	100	77
	10	60	5	63	87	100	72
	11	79	8	48	91	100	63
	12	84	4	45	73	100	55
	13	60	5	38	78	64	48
	14	68	4	26	45	100	37
	15	69	26	2	28	0	9
	16	71	18	6	10	10	8
	17	66	3	9	1	ND	7
	18	52	7	4	5	ND	4
		65 ± 11^f^	8 ± 6^f^	59 ± 37^f^	70 ± 36^f^	84 ± 33^f^	65 ± 41^f^
**VV1**	1-5	32 ± 5^f^	11 ± 2^f^	0	0	0	0

^a^Expressed as individual numerical values or means ± SD. ^b^All T2% values determined by densitometry on long gels WB probed with Ab 3F4 and 1E4. ^c^Average of frontal (superior and middle gyrus), temporal, parietal, occipital (visual and non-visual), entorhinal and hippocampal cortices. ^d^Average of putamen, thalamus and substantia nigra. ^e^Average of previous three brain regions. ^f^Expressed as mean ± standard deviation. ND: Neither T1 nor T2 were detected.

### Western blot profile and brain regional distribution of resPrP^D^

**Figure 1:**
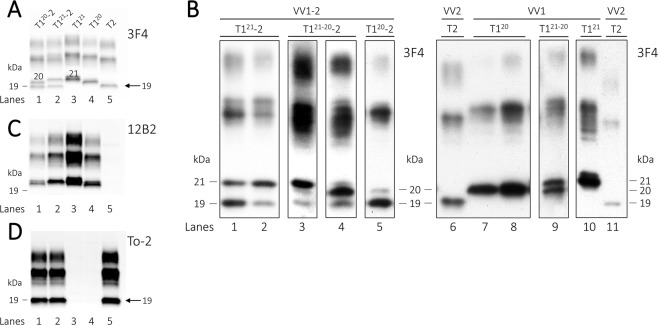
Western blot profiles of resPrP^D^. Supernatants (S1) (See Materials and Methods) from the cerebral cortex were treated with 10 U/ml PK, probed with various Abs and visualized using the near-infrared LI-COR system and 8.7 cm-long gels (**A**,**C** and **D**) or chemiluminescence using 20 cm-long gels (**B**). (**A**) In sCJDVV1–2 (lanes 1 and 2) and -VV1 (lanes 3 and 4), the unglycosylated isoforms of the T1 variants migrated as a predominant band of ~20 kDa (lanes 1 and 4) or of ~21 kDa (lanes 2 and 3); in -VV2 (lane 5), T2 migrated to ~19 kDa (arrow). (**B**) High resolution WB profiles of T1, T2 and T1–2. In sCJDVV1–2 (left panel), T1–2 features the ~21 and ~20 kDa bands occurring separately (lanes 1, 2 and 5) or together in different proportions (lanes 3 and 4) along with variable amounts of the ~19 kDa band representing T2 resPrP^D^ (lanes 1–5). In sCJDVV1 (right panel), T1 formed bands of ~20 kDa (lane 7 and 8), a doublet of ~21–20 kDa (lane 9), or a band of ~21 kDa (lane 10). sCJDVV2 (lanes 6 and 11) is used as T2 control. Each box represents cropped parts of different WB (see Figure S2 for the uncropped WB). Cerebral cortex (lanes 1, 2, 5–8, 11), subcortical regions (lanes 3, 4, 9), cerebellum (lane 10). (**C**) 12B2 (to T1) immunoreacts with the ~21 and ~20 kDa fragments (lanes 1–4), but not with T2 (19 kDa) (lanes 1, 2 and 5). (**D**) To-2 reacts with T2 (arrow; lanes 1, 2 and 5).

The Western blot (WB) profile of -VV2 was typical for T2 resPrP^D^ as it showed the unglycosylated form with the electrophoretic mobility of ~19 kDa as previously reported (Fig. 1)^1,10^. By contrast, in -VV1, T1 comprised two variants, commonly identified by the unglycosylated isoforms as ~20 kDa (T1^20^) and ~21 kDa (T1^21^) respectively, both of which immunoreacted with the T1-specific Ab 12B2 (Fig. 1C)^11^. The two T1 variants could be seen either together as a ~20–21 kDa doublet, where they coexisted in different ratios, or separately (Fig. 1B).

In -VV1–2, the T1^20^ and T1^21^ components formed a doublet in 44% of the samples examined or occurred separately in 29% (T1^21^) and 27% (T1^20^). T1^21^ predominated over T1^20^ in each brain compartment examined since it accounted for 69% in the cerebral cortex, 78% in subcortical regions and 65% in cerebellum. Moreover, based on the examination of 17 -VV1–2 cases, we noted a possible overall inverse relation between the relative amounts of T1^21^ and T2. As expected, resPrP^D^ T2 was strongly recognized by To-2 in both -VV2 and -VV1–2 (Fig. 1D).

**Figure 2:**
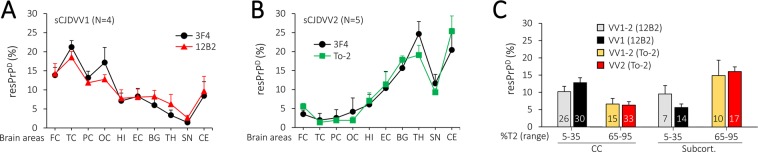
Brain distribution of T1, including T1^20^ and T1^21^, and T2. (**A**,**B**) Quantitative distribution profiles of T1 and T2 in the indicated brain anatomical regions of -VV1 (**A**) and -VV2 (**B**). Each point of the profile is expressed as mean ± SEM; FC, TC, PC, EC, VC, OC: frontal, temporal, parietal, entorhinal, visual and occipital cortex; HI: hippocampus; BG: basal ganglia; TH: thalamus; SN: substantia nigra; CE: cerebellum. (**C**) Relative amounts of T1 and T2 in cerebral cortex (CC) and subcortical regions (Subcort.) of -VV1–2 mirrored those of -VV1 and -VV2, respectively; numbers under the ordinate indicate the percent amount of T2 implying that T1 accounts for remaining percentage (not shown); numbers embedded in the bar graphs indicate sample size. VV1 vs. VV2, P < 0.0003 (CC) and P < 0.0001 (Subcort.).

In agreement with previous studies, the rostrocaudal quantitative distribution of resPrP^D^ in -VV1 and -VV2, examined with generic and type-specific Abs, was virtually inverse where most resPrP^D^ occupied the cerebral cortex in -VV1, as opposed to the subcortical regions including the cerebellum in -VV2 (Fig. 2A,B)^12^. In sCJDVV1–2, T2 predominated over T1 in the cerebellum as it accounted for 84% of the total resPrP^D^; furthermore, 11 of the 16 cases examined harbored exclusively T2. In contrast with the cerebellum, T2 relative amounts in cortical and subcortical regions were 59% and 70%. Additionally, over 95% of T2 occurred in 3 cerebral cortical and 7 subcortical regions of the 18 cases examined (Table 1). These findings suggest that in -VV1–2, T2 distributed according to a gradient that increased from cerebral cortex to subcortical nuclei and cerebellum (Table 1). To determine whether the regional distribution of resPrP^D^ T1 and T2 in -VV1–2 mimicked the distributions characteristic of -VV1 and -VV2 according to whether T1 or T2 were the dominant component, we divided the -VV1–2 cases into two groups with low (5–35%) and high (65–95%) relative amount of T2 (Fig. 2C). T1 and T2 percent distribution was determined in cerebral cortical and subcortical regions (excluding the cerebellum that was populated almost exclusively by T2). The -VV1–2 subset with low T2 presence showed a T1 distribution that did not significantly differ from the T1 distribution in -VV1. Likewise, when highly represented, T2 in -VV1–2 showed a -VV2-like distribution pattern (Fig. 2C).

### PrP^D^ conformational assays

PK-titration assay and conformational solubility and stability assay (CSSA) were applied either to resPrP^D^ alone or to totPrP^D^ (PK-sensitive PrP^D^ + resPrP^D^) associated with T1 and T2 in -VV1–2 to assess conformational features, which are helpful to identify distinct strains.

**Figure 3:**
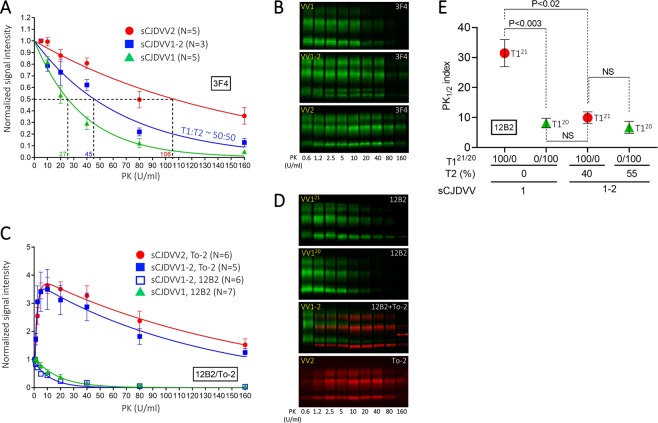
PK-titration assay. Supernatant (S1) from each of the three sCJDVV subtypes was digested with PK concentrations of 0.6 to 160 U/ml, as indicated, and probed with 3F4 (**A**,**B**), 12B2 and To-2 (**C**,**D**) or 12B2 (**E**). (**A**) PK_1/2_ (index denoting amount of PK in Units/ml required to digest half of resPrP^D^) for sCJDVV1–2 (45 ± 3 U/ml) was about intermediate between that of T1 in -VV1 (27 ± 4 U/ml) and that of T2 in -VV2 (106 ± 13 U/ml) (PK_1/2_ VV1 vs. VV2, P < 0.0006; PK_1/2_ VV1–2 vs. VV1, P < 0.03; PK_1/2_ VV1–2 vs. VV2, P < 0.02). (**B**) Representative WB of resPrP^D^ obtained at the indicated PK concentrations from each of the three sCJDVV subtypes and used to generate the curves of (**A**,**C**) S1 from the three sCJDVV subtypes probed with the T1- and T2-specific Abs 12B2 and To-2. Hydrolysis profiles of T1 and T2 from sCJDVV1–2 mimic those of T2 from -VV2 and T1 from -VV1, respectively. PK_1/2_ in T1 from -VV1 and -VV1–2 are 18 ± 5 U/ml and 8.3 ± 1.5 U/ml, respectively (P > 0.05); PK_1/2_ in T2 from -VV2 and VV1–2 are 130 ± 15 U/ml and 142 ± 26 U/ml, respectively (P > 0.05). The peculiar double exponential equation profile observed with To-2 likely relates to the specific and efficient detection by this Ab of PrP residue 97 only when this residue is exposed as N-terminus; this condition requires a distinct initial digestion phase^20^. (**D**) Representative WB of T1 (12B2; green dye) and T2 (To-2; red dye) obtained at the different PK-concentrations in the three sCJDVV subsets. (**E**) PK_1/2_ determination of T1^21^ and T1^20^ in the absence and presence of T2. A significant reduction in PK-resistance occurs in T1^21^ in the presence of T2 (PK_1/2_ 31.5 to 10 U/ml) while T1^20^ maintains the comparable PK_1/2_ (8.2 and 6.7 U/ml).

The PK-titration assay was carried out with the type-generic Ab 3F4 to probe resPrP^D^ from -VV1–2 harboring approximately equal amounts of T1 and T2. The PK_1/2_ index (amount of PK needed to hydrolyze 50% PrP^D^) was 45 U/ml, which was roughly intermediate between, and significantly different from, the -VV2 (106 U/ml) and -VV1 (27 U/ml) indexes (Fig. 3A,B). Type-specific Abs, which separated the titration curves of T1 and T2 associated with -VV1–2, demonstrated that the PK high resistance curves were populated by T2 and those with low resistance by T1. Remarkably, the two curves generated by -VV1–2 mirrored the curves of -VV1 and -VV2, respectively (Fig. 3C,D). This finding further underscores the commonality of the -VV1–2 T1 and T2 with the T1 and T2 associated with -VV1 and -VV2. However, when the T1^21^ and T1^20^ variants were tested separately, T1^21^ revealed a significantly higher PK-resistance than T1^20^ (PK_1/2_ indexes of 31.5 and 8.2 U/ml, respectively). Furthermore, in -VV1–2 cases harboring significant amounts of T2, T1^21^ PK_1/2_ decreased ~3.2 fold to 10 U/ml matching the T1^20^ index that remained essentially unchanged (Fig. 3E).

**Figure 4:**
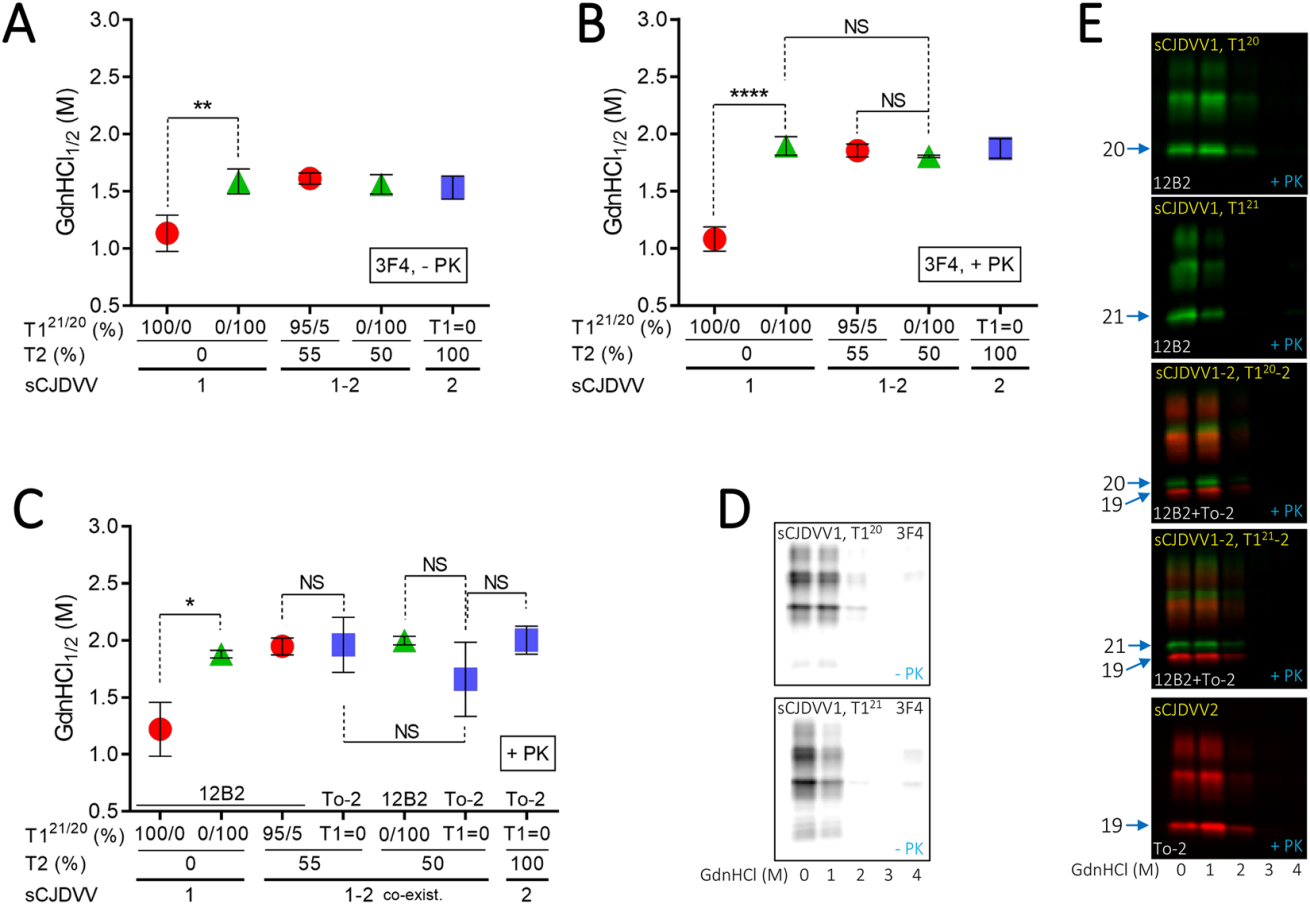
Conformational solubility and stability assay (CSSA). GdnHCl_1/2_ quantity of GdnHCl required to solubilize half of totPrP^D^, (**A**,**D**), or of resPrP^D^, (**B**,**C,E**). (**A**,**B**) GdnHCl_1/2_ indexes were qualitatively similar for totPrP^D^ (**A**) and resPrP^D^ (**B**). In -VV1, T1^21^ was significantly less stable than T1^20^ while in -VV1–2 cases harboring T2 in significant amounts, T1^21^ showed a GdnHCl_1/2_ similar to that of T1^20^, both mimicking the GdnHCl_1/2_ of T2. (**C**) GdnHCl_1/2_ index obtained after probing resPrP^D^ with the type-specific Abs 12B2 to T1 and To-2 to T2 were comparable with those generated using the type-generic Ab 3F4 in **A** and **B**. (**D**,**E**): Representative WB of totPrP^D^ from -VV1 (**D**), and resPrP^D^ from each sCJDVV subtype (**E**). *P < 0.05–0.04, **P < 0.02, ****P < 0.0006–0.0001. sCJDVV1 with T1^20^ (N = 3) or T1^21^ (N = 3); -VV1–2 with T1^20^ (N = 3) or T1^21^ (N = 4), -VV2 (N = 3).

The CSSA, which allows for the examination of the PK-sensitive portion of PrP^D^ (senPrP^D^) in addition to resPrP^D^ (senPrP^D^ + resPrP^D^ = totPrP^D^), revealed similar indexes for totPrP^D^ and resPrP^D^ alone. Probing totPrP^D^ with the type-nonspecific Ab 3F4, GdnHCl_1/2_ indexes (GdnHCl molar concentration required to solubilize half of totPrP^D^) related to T1^21^ and T1^20^ significantly differed from each other (-VV1, T1^21^ = 1.13 M; T1^20^ = 1.59 M). However, in -VV1–2 regions where T1^21^ and T1^20^ coexisted with significant amounts of T2, the T1^21^ index increased matching the indexes of T1^20^ and T2 (T1^21^ = 1.61 M; T1^20^ = 1.56 M; T2 = 1.53 M) (Fig. 4A,D). Similar results were observed with resPrP^D^ (-VV1, T1^21^ = 1.08 M and T1^20^ = 1.89 M). Again, in -VV1–2 regions where T1^21^ and T1^20^ coexisted with T2, the T1^21^ index increased matching the indexes of T1^20^ and T2 (T1^21^ = 1.85 M; T1^20^ = 1.80 M; T2 = 1.87 M) (Fig. 4B). Similar indexes were acquired when probing the same resPrP^D^ substrates with the type specific Abs (Fig. 4C,E). Collectively, these data indicate that T2 has the same conformational indexes in -VV2 and -VV1–2; however, stability characteristics of T1^21^ (as determined by GdnHCl_1/2_) changed in the presence of ~50% T2, becoming comparable to the stability characteristics of T1^20^ and T2. Therefore, T2 presence would influence conformational features of T1^21^.

### Phenotype characterization: histopathology, immunohistochemistry, and lesion profiles

**Figure 5:**
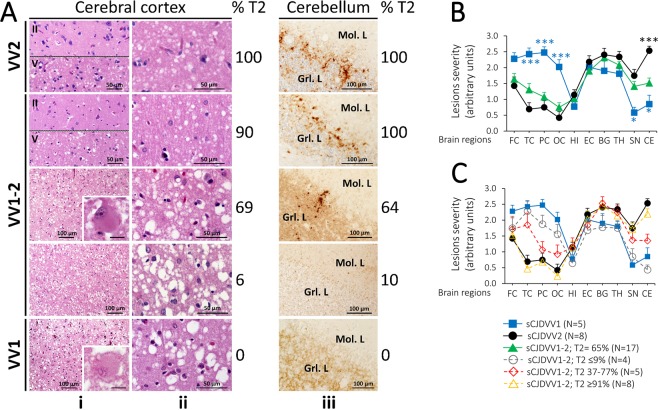
Histopathological phenotypes and lesion profiles. (**A**) Hematoxylin and eosin (**i** and **ii**) and PrP immunohistochemistry (**iii**). **i**-**iii** (1^st^ and 2^nd^ rows), **i**: Spongiform degeneration (SD) affecting only deep cortical layers (II: layer 2; V: layer 5); **ii**: Small vacuoles SD; **iii**: Plaque-like PrP immunostaining in the granular layer (Grl. L.) of the cerebellum; Mol. L: molecular layer; **i**-**iii** (3^rd^-5^th^ rows); **i**: SD with medium size vacuoles, scattered ballooned neurons and atrophy affecting all layers of the cerebral cortex; insets in **i** (rows 3^rd^ and 5^th^): a ballooned neuron. **iii**: Presence and lack (3^rd^, 4^th^ and 5^th^ rows, respectively) of plaque-like PrP in the granular layer. Scale bar insets: 25 µm. (**B**,**C**) Lesion profiles. (**B**) The lesion profile of all -VV1-2 cases combined resembled that of -VV2 except for the cerebellum (CE). (**C**) Lesion profiles of -VV1-2 with low (≤9%; cases 15-18, Table S2), intermediate (37–77%; cases 9-11, 13, 14) and high (≥91%; cases 1-8) representation of T2. The -VV1-2 lesion profile shifts progressively from resembling -VV1 at low %T2 to virtually coincide with -VV2 at high %T2. Each point of the lesion profile is expressed as mean ± SEM of severity scores related to spongiosis and gliosis. FC: frontal cortex (cx); TC: temporal cx; PC: parietal cx; OC: occipital cx; HI: hippocampus (CA1 region); EC: entorhinal cortex; BG: basal ganglia; TH: thalamus, SN: substantia nigra; CE: cerebellum. *P < 0.05-0.04; ***P < 0.0006-0.0001.

Overall, the histopathological phenotype associated with -VV1–2 showed regional features of -VV1 and -VV2 that were directly related to the relative amounts of T1 and T2 (Fig. 5). Cerebral cortical histopathology with -VV1 features was detected in -VV1–2 cases where cortical T2 accounted for 69% or less (cases 9–18, cerebral cortex data in Table S2 and Fig. 5A). In -VV1–2, as in -VV1, no histopathological features specifically associated with the presence of either T1^21^ or T1^20^ were identified. Conversely, spongiform degeneration (SD) with laminar distribution, which is typical of -VV2, was invariably present in the -VV1–2 cases harboring 87% or more T2 (see cerebral cortex data, cases 1–8 in Table S2, and Fig. 5A), and it was observed only in 3 out of 10 of the -VV1–2 cases harboring 69% or less T2 (cases 10, 12 and 14 in Table S2). In the cerebellum, the study of lesion burden in sCJDVV1–2 was problematic because cerebellar T2 exceeded 64% in the majority of our cases (cases 1–14 cerebellum data in Table S2). However, atrophy and plaque-like PrP immunostaining pattern of the granule cell layer, the two most consistent -VV2 lesions, were lacking or minimal in the two cases where cerebellar T2 was 10% or undetectable (cases 16 and 18 cerebellum data in Table S2, Fig. 5A).

With the exception of the cerebellum, the overall lesion profile of -VV1–2 resembled that of -VV2 (Fig. 5B). However, when -VV1–2 cases were separated into three groups according to T2 relative amounts, that is ≤9% (N = 4, cases 15–18), ≥91% (N = 8, cases 1–8) and 37–77% (N = 5, cases 9–14), as expected, in the presence of ≤9% and ≥91% T2, -VV1–2 lesion profiles mimicked those of -VV1 and -VV2, respectively; when the T2 amounts were intermediate (37%-77%), the lesion profile fit roughly halfway between those of -VV1 and -VV2 (Fig. 5C, Tables 1 and S2).

The -VV1–2 case cohort was divided into three groups according to T2 relative amount in the cerebral cortex (≥87%, cases 1–8; 26–69%, cases 9–14; ≤9%, cases 15–18) (Tables 1 and S2). VV1-like pathological features, which were absent in -VV2, increased progressively and inversely correlated with the relative amounts of T2 through the three -VV1–2 subsets (r = −0.96–0.99). A similar but direct correlation was observed for the presence of laminar SD (r = 0.99) (Table S2) as well as in the cerebellum where both atrophy (r = 0.93) and plaque-like PrP staining (r = 0.99) similarly correlated with T2 relative amounts (Table S2).

### Clinical features and prevalence of sCJDVV1, -VV2, and -VV1–2

The sCJDVV1–2 case cohort was divided into three groups according to T2 relative amount (≥91%, cases 1–8; 37–77%, cases 9–14; ≤9%, cases 15–18), and the clinical features of each group, either individually or combined, were compared with those of -VV1 and -VV2 (Table 2). The -VV1–2 mean age at onset did not significantly change as a function of the T2 amount, and in the three -VV1–2 groups combined (65 ± 11 y) was comparable to that of -VV2 (68 ± 8 y) while differing significantly from that of -VV1 (32 ± 5 y) (P < 0.0001). Disease duration in sCJDVV1–2 inversely correlated with T2 percentage (r = −0.88) but it was identical to that of -VV2 in the  ≥ 91% and 37%-77% T2 groups while the ≤9% group showed a duration comparable to that of -VV1 (Table 2).

**Table 2:** Selected clinical features of sCJDVV1-2, sCJDVV1 and sCJDVV2.

Presentation	sCJDVV2	sCJDVV1-2			sCJDVV1
Case number (Table 1)	1-8	1-8	9-14	15-18	1-5
% T2	100	≥91	37-77	≤9	0
Age at onset (years) (mean ± SD; median) [range]^a^	68 ± 8; 72 [56–78]	62 ± 12; 68 [44–74]	68 ± 11; 64 [57–84]	64 ± 9; 67 [52–71]	32 ± 5; 34 [25–38]
Duration (months) (mean ± SD; median) [range]^b^	6 ± 2; 5 [3–9]	6.5 ± 4; 5 [3–17]	6 ± 2; 5 [4–8]	13.5 ± 10; 12 [3–26]	11 ± 2; 12 [8–14]
Male gender	38^c^ (3/8)^d^	50 (4/8)	67 (4/6)	25 (1/4)	60 (3/5)
Cognitive decline^e^	33 (2/6)	37 (3/8)	67 (4/6)	100 (3/3)	100 (4/4)
Cerebellar signs^f^	100 (6/6)	88 (7/8)	50 (3/6)	0 (0/3)	0 (0/4)
PSWC on EEG	0 (0/6)	20 (1/5)	33 (1/3)	0 (0/4)	0 (0/4)
Positive 14-3-3	100 (6/6)	100 (8/8)	100 (4/4)	100 (2/2)	100 (3/3)

^a^All sCJDVV1-2 cases combined and each of the three groups indicated were significantly different (P < 0.0004-0.0001) from -VV1 but not from -VV2 cases. ^b^VV1-2 cases 1-8 and 9-14, combined were significantly different from VV1 (P < 0.007); cases 15-18 were not significantly different from VV1. ^c^Expressed in percent; ^d^cases with the feature listed/total cases examined. ^e,f^Cognitive decline and cerebellar signs were respectively inversely and directly correlated to % T2 (r = −0.99 and r = 0.99). Statistical significance was calculated using Student’s t-test for age and duration, and Fisher’s exact test to determine non-random associations in the other clinical data; Pearson’s test was used to assess correlations; SD: standard deviation.

Among the clinical signs, cognitive decline and cerebellar signs showed a strong opposite correlation with the T2 relative amount. Cognitive decline, present in 33% (2/6) of the patients with -VV2, increased progressively, and inversely correlated, through the three -VV1–2 groups becoming present in all patients as in -VV1, paralleling the T2 decrease from ≥91% to ≤9% (r = −0.99). A similar but direct correlation with T2 percentage was also observed for the cerebellar signs that progressively decreased through the three groups from 100% to 0% (r = 0.99) (Table 2).

To assess demographics and prevalence on a larger sample of sCJDVV1–2 cases, we examined 463 consecutive cases of sCJDVV that received a definitive diagnosis of subtype at the NPDPSC between 2002 and 2017. According to NPDPSC standard diagnostic procedures, which included WB examination of 1 to 3 brain regions (frontal, occipital and cerebellar), 8% (N = 35) of the cases were diagnosed with sCJDVV1, 69% (N = 320) with -VV2 and 23% (N = 108) with -VV1–2. The sCJDVV1–2 diagnosis was based on the examination of three brain regions in 88% of the cases. The mean age at onset in -VV1–2 (64 ± 10 y) resembled that of -VV2 (66 ± 9 y), differing significantly from that of -VV1 (54 ± 13 y) (P < 0.0001); similarly, disease duration (6.6 ± 7.5 mo) was not significantly different (P = 0.07) from that of -VV2 (5 ± 3 mo), but significantly differed from that of -VV1 (10 ± 7 mo) (P < 0.02). The 23% prevalence of sCJDVV1–2 according to standard NPDPSC diagnostic procedure rose to 57% when the same stringent criteria followed to select the 18 sCJDVV1–2 cases used in this study were applied to the 463 consecutive cases with sCJDVV. Also the discrepancy as to age at onset and disease duration between the standard and rigorously selected sCJDVV1 populations is likely to be due to the presence of -VV1–2 cases (associated with low amounts of PrP^D^ T2) in the -VV1 cohort extracted from the standard NPDPSC consecutive cases.

## Discussion

A major finding of the present study is that the histopathological features present in sCJDVV1–2 mimic those of -VV1 or -VV2, with the lesion burdens directly related to the relative amount of resPrP^D^ T1 and T2, respectively. Considering that similar results were observed in previous studies of sCJDMM1–2^4,6^, sCJD type-mixed cases can be defined as subjects in which i) resPrP^D^ T1 and T2 co-exist in ratios that may vary widely in different brain regions, and ii) histopathological features are typically associated with the dominant resPrP^D^ type. Combined, these studies strongly support the correlation between PrP^D^ type and histopathological phenotype as originally proposed by Parchi *et al*.^1^. The correlation between T1:T2 ratio and clinical features was less consistent except for cognitive decline and cerebellar signs, two distinctive clinical features of -VV1 and -VV2, respectively. In VV1–2, these two signs were highly correlated with the T1:T2 ratios whereas age at onset and disease duration mostly mimicked -VV2 figures. Although the analysis of clinical data was less detailed, opposite findings were found in sCJDMM1–2 where only disease duration but none of the clinical signs was related to the T1:T2 ratios^4^.

The almost threefold difference between the 23% prevalence of sCJDVV1–2 that we observed following standard diagnostic procedures of sCJD subtype and the 57% prevalence following the more stringent conditions of this study is not surprising given the extensive sampling and sensitive detection procedure adopted. For practical diagnostic purposes, this difference may also be of limited consequence since below 25% the underrepresented resPrP^D^ type carries virtually no weight in determining the disease phenotype.

Another remarkable feature of sCJDVV1–2 is the preservation of the original rostrocaudal distributions that T1 and T2 originally display in -VV1 and -VV2, respectively^12,13^. Accordingly, the relative amounts of the T1 component tended to be higher in the cerebral cortex while the T2 clearly dominated in the subcortical regions and cerebellum. A similar preferential distribution of T1 and T2 was observed in sCJDMM1–2 where T2 relative presence prevailed in the cerebral cortex to become underrepresented in the cerebellum^4,6^. However, in sCJDVV1–2, overall T2 appeared to be better represented than T1 while the opposite was true for -MM1–2^4,6^. Collectively, these findings indicate that the characteristic distribution pattern of each resPrP^D^ type is maintained also in the presence of a second type, and suggest that the overall resPrP^D^ type prevalence in T1–2 mixed cases reflects the prevalence of the original diseases in which -VV2 and -MM1 are by far more prevalent than -VV1 and -MM2; this type-prevalence in mixed cases may reflect the higher propensity to reproduce and propagate of the PrP^D^ dominant type^14–16^.

The study of sCJDVV1–2 was further complicated by the presence of two T1 variants, identified as T1^21^ and T1^20^ (originally described by Notari *et al*.^11^). Using high resolution gel electrophoresis under stringent conditions, T1^21^ and T1^20^ were seen together as a doublet, or separately in both -VV1–2 and -VV1. This pattern can be considered the distinguishing feature of the T1 variant associated with sCJDVV since T1^20^, but not T1^21^, is detected in -MM1 under the same methodological conditions^4^. T1^21^ and T1^20^ electrophoretic mobility and the immunoreactivity with Abs T1-specific 12B2 raised to PrP residues 89–93, but not with T2-specific To-2 to residues 97–103, are consistent with N-termini exclusively associated to resPrP^D^ type 1. N-terminus sequencing of two cases of sCJDVV1 prior to the T1^21^ and T1^20^ discovery, showed a major N-terminus at residue 82, consistent with the predominant T1^21^ and minor termini at residues 86 and 90, which might identify T1^20^ since the latter two termini were not detected in sCJDMM1 T1 preparations^17^; only N-terminus sequencing will resolve this issue. Although no histopathological feature was specifically associated with these two VV T1 variants, conformational tests showed that T1^21^ and T1^20^ have significantly different PK-resistance and stability characteristics, suggesting that they are distinct strains. Furthermore, even though T1^21^ showed the same conformational characteristics in sCJDVV1 and -VV1–2 when it was harbored in the brain regions lacking T2, these characteristics significantly shifted in the presence of significant amounts of T2.

In the study of sCJDMM1–2, we reported that the stability characteristics, established by the conformational stability immunoassay (CSI) based on chemiluminescence, significantly changed in T1 when significant amounts of T2 coexisted. The intriguing possibility that conformational characteristics of a PrP^D^ T1 or T2 might change when co-existing with PrP^D^ of a different type as well as whether T1^21^ and T1^20^ are distinct strains despite being associated with the same phenotype, need to be confirmed with more advanced conformational tests and, ultimately, by bioassay.

The early and accurate clinical diagnosis of sCJD type-mixed patients is especially challenging. RT-QuIC (real time-quacking induced conversion), a common and reliable test for the diagnosis of prion disease based on the detection of minute amounts of PrP^D^ following amplification by conversion, apparently has the capability of distinguishing individual major subtypes of sCJD^8^; however, it fails to identify sCJD mixed subtypes due to the selective amplification of the PrP^D^ component that is more easily replicated. Recently, diffusion MRI (dMRI) has also been shown to allow for early and reliable diagnosis of sCJD subtypes (Bizzi *et al*., submitted). The dMRI diagnosis is based on the distinct profile of the dMRI signal generated by the cerebral cortex and subcortical nuclei by each sCJD subtype, roughly reflecting the severity and anatomical distribution of the underlying histopathology. The propensity of state-of-the-art dMRI to identify individual sCJD type mixed cases has not been assessed. Since virtually all mixed cases with the approximate <25% relative amount of one of the T1 and T2 types actually display only one histopathological phenotype, the mixed nature of this subset, which accounted for approximately 70% of cases in our study, will likely remain undetected by dMRI. The identification of the remaining cases harboring two histopathological phenotypes may require significant improvements in the dMRI resolution allowing for the detection of SD with distinct vacuolar sizes and affecting distinct cortical layers. Finally, whether the co-occurrence of even small relative amounts of a second type of PrP^D^ will affect future therapeutics remains to be determined.

## Materials and Methods

See Supplementary Materials and Methods for the reagents and antibodies used, and for the description of the following: i) brain homogenate preparation, ii) WB analysis, iii) molecular genetics, iv) brain regional distribution of resPrP^D^, v) conformational solubility and stability assay, vi) clinical evaluation, and vii) image acquisition and statistical analysis.

### Subjects and ethics statement

We selected a cohort of 31 patients, all of whom were valine homozygous at codon 129 (129VV) of the human PrP gene, as determined by the definitive diagnosis of sCJD (sCJDVV) made at the NPDPSC in Cleveland, OH, USA. This cohort had undergone standard WB examination of three brain regions (frontal and occipital cortices, and cerebellum) at the NPDPSC. We then performed a detailed analysis of 12 brain regions under stringent experimental conditions (see below) in each of the 31 sCJDVV cases, which resulted in the following case distribution: (i) cases classified as sCJDVV1 (N = 5) that harbored exclusively resPrP^D^ type 1 (T1); (ii) sCJDVV2 cases (N = 8) harboring exclusively resPrP^D^ type 2 (T2); (iii) mixed types harboring both types 1 and 2 (T1–2) (sCJDVV1–2, N = 18). Patients lacked pathogenic mutations in *PRNP* and had no history of familial diseases or known exposure to prion agents.

All procedures were carried out under protocols approved by the Institutional Review Board (IRB) at Case Western Reserve University. Written informed consent for research was obtained from all patients or legal guardians according to the Declaration of Helsinki. All patients’ data and samples were coded and handled in accordance with NIH guidelines to protect patients’ identities.

### Brain sampling

Coronal sections obtained at autopsy from one half brain and stored at −80 °C were used for the molecular studies. Samples from the other formalin-fixed half were used for histology and immunohistochemistry. For WB, we planned to sample 12 brain regions from each case: frontal (superior and middle gyrus), temporal, parietal, visual and non-visual occipital neocortices, hippocampus (CA1-CA4), entorhinal cortex, basal ganglia (putamen), thalamus, substantia nigra and cerebellum. Of the 372 brain regions to be sampled from the 31 sCJDVV cases combined, 25 were not available. Of these 25, 6 were from sCJDVV2 [3 each from cerebral cortex (CC) and subcortical regions (scr)]; 17 from sCJDVV1–2 (10 from CC and 7 from scr); 2 from sCJDVV1 (1 each from CC and cerebellum). Moreover, no resPrP^D^ signal either for T1 or T2 was detected in 6 samples from 4 sCJDVV cases including 3 sCJDVV1–2 (3 from CC; 2 from cerebellum) and one sCJDVV1 (from CC). Therefore, a total of 341 brain regions were examined for this study.

### Histology, PrP immunohistochemistry, and lesion profiles

Formalin-fixed brain tissue was treated as previously described^18^. Briefly, sections were deparaffinized and rehydrated, immersed in 1X Tris buffered saline containing Tween 20 (TBS-T), and endogenous peroxidase blocked after incubation with the Envision Flex Peroxidase Blocking Reagent for 10 minutes (min). Sections were washed, immersed in 1.5 mmol/L hydrochloric acid, microwaved for 15 min and incubated with the Ab 3F4 (1:1,000) for 1 hour (h). After washing and incubation with Envision Flex/HRP polymer for 30 min, sections were treated with Envision Flex DAB for immunostaining. Histological sections from ten brain regions, which included frontal, temporal, parietal, occipital and entorhinal cortices as well as the hippocampus, basal ganglia, thalamus, substantia nigra and cerebellum, were examined to evaluate severity and distribution of spongiform degeneration and gliosis according to previous studies^1,19^. Severity of SD and gliosis was scored on a 0 to 4 scale (not detectable, mild, moderate, severe, and status spongiosus), and on a 0 to 3 scale (not detectable, mild, moderate, and severe), respectively. Lesion severity scores of SD and gliosis were averaged in each brain region and expressed as mean ± SEM.

Qualitative assessment of histopathological changes included the determination of: 1) vacuole size (small vs. medium size vacuoles); 2) presence of ballooned neurons; 3) pattern of SD in the CC (laminar, affecting the deep layers vs. full thickness, affecting all cortical layers); 4) atrophy of the cerebellar granular layer. Immunohistochemistry was carried out to determine the distribution of PrP deposition in the CC (laminar vs. full thickness) and cerebellum (i.e., plaque-like PrP deposits in granule cell layer).

### Optimal PK-concentration

To rule out the possibility that the ~20 kDa fragment was a product of incomplete PK digestion, supernatants (S1) from sCJDVV2 and -VV1 were digested with increasing concentrations of PK and probed with the T1-specific Ab 12B2. In these experiments, the 12B2-immunoreactive resPrP^D^ underwent proteolysis that was much faster in -VV2 than in -VV1, with resPrP^D^ signal detectable with up to ~ 2.5–5 U/ml in -VV2 (N = 3) and ~ 40–80 U/ml in -VV1 (N = 4). The concentration of PK needed to reduce up to 50% of the initial PrP^D^ amount, the PK_1/2_ index, was 17-fold lower in -VV2 than -VV1 (0.4 U/ml vs. 6.9 U/ml; P < 0.004) (Supplementary Figure S1A,B). In these experiments, PK_1/2_ was calculated by fitting a one-phase decay equation to the experimental PK points ranging between 0.6 to 160 U/ml. For large scale validation of these results, various brain regions from eight -VV2 cases were tested with 12B2. For this experiment, two aliquots of the same sample were digested with 0.6 and 10 U/ml PK using equal loading volumes. While this low PK concentration was used to normalize the intensity of resPrP^D^ signal from all samples, 10 U/ml PK was adopted to discriminate between the *bona fide* T1 and partially cleaved fragments. As shown in Supplementary Figure S1C, resPrP^D^ was not visualized by 12B2 in samples treated with 10 U/ml PK whereas it was after 0.6 U/ml. We used 10 U/ml PK dosage to assess the T1:T2 ratio.

### Antibodies selection

The percentage ratio of T1 and T2 unglycosylated isoforms of resPrP^D^ co-existing in the same anatomical region or found separately in the same brain of sCJDVV1–2 cases, as well as the exclusive presence of T1 or T2 in -VV1 and -VV2 was established probing with Ab 3F4 and 1E4. In the -VV1–2 subtype, 3F4 revealed T1–2 in 107 of 142 (75%) brain regions examined (while 52 regions had T2 only). Following 1E4 immunoblotting of 35 brain regions that on WB probed with 3F4 were populated exclusively by T1, the co-existence of T2 was demonstrated in a total of 129 (91%) brain regional samples (16% increase).

### PK-titration assay

S1 were digested with PK at concentrations of 0.6, 1.2, 2.5, 5, 10, 20, 40, 80, and 160 U/ml at 37 °C for 1 h, and the reactions stopped with 3 mM PMSF. In -VV1, the PK points were best fitted by a one-phase decay equation, while in -VV1–2 and -VV2 they were best fitted by a one-phase decay or double exponential equation depending on whether 3F4 or To-2 was employed. In experiments with 3F4, PK_1/2_ index was calculated by fitting a one-phase decay to PK points ranging between 5 to 160 U/ml. The reason for excluding the lower PK concentration points (e.g., 0.6, 1,2 and 2.5 U/ml) was due to the fact that T2 in -VV1–2 and -VV2 showed a different behavior compared to T1, making the fitting into a one-phase decay curve or other types of curves poor (i.e., low values of R^2^). In experiments using To-2, PK_1/2_ index values were determined considering the part of the profile that decayed (i.e., PK-concentration points ranging from 5 to 160 U/ml). The portion of the profile in the PK-range 0.6–2.5 U/ml was indistinguishable in -VV2 and -VV1–2. The PK_1/2_ index values were determined with GraphPad Prism 8.1.1 and were expressed as mean ± SEM.

### Prevalence determination of sCJDVV subtypes following standard and strict procedures

Review of sCJD cases examined at the NPDPSC revealed 463 consecutive cases of sCJDVV that received a definitive diagnosis of subtype between 2002 and 2017. Of these cases, 35 (8%) were sCJDVV1, 320 (69%) sCJDVV2 and 108 (23%) sCJDVV1–2. All cases were diagnosed following the NPDPSC typical procedure which includes standard WB examination of 1–3 brain regions (frontal and occipital cortices, and cerebellum) using the Ab 3F4 supplemented with Abs 12B2 and 1E4. Of the 108 -VV1–2 cases, 68 (63%) were probed with 3F4 only, 31 (29%) with 12B2 and 9 (8%) with 1E4, besides 3F4; furthermore, 88% (N = 95) of these cases underwent WB examination of all three regions (in 7% and 5% only two and one regions were available). Routine diagnosis of sCJDVV1–2 in the 463 NPDPSC cases was made when both 19 kDa and 20–21 kDa bands were visually detectable in any of the three brain regions. To determine the prevalence of sCJDVV1–2 in the 463 sCJDVV case cohort according to the “stringent” procedure, we applied to the 463 sCJDVV cases the same numerical corrections followed to select the 18 -VV1–2 cases of the detailed study (Table 1, Subjects and Brain sampling above). These stringent procedures resulted in the inclusion in the sCJDVV1–2 subtype of cases that harbored downwards to 1% of either T1 or T2 resPrP^D^ expressed as average of all brain samples examined.

## Supplementary information

Supplementary Information
