# Supplementary material for: Co-existence of PrPD types 1 and 2 in sporadic Creutzfeldt-Jakob disease of the VV subgroup: phenotypic and prion protein characteristics
Source: Sci Rep. 2020 Jan 30;10:1503. doi: 10.1038/s41598-020-58446-0 (PMC6992672; doi:10.1038/s41598-020-58446-0)
Supplement: Supplementary file 1 — Supplementary Information [file 41598_2020_58446_MOESM1_ESM.pdf]

SUPPLEMENTARY INFORMATION

Co-existence of PrP^D types 1 and 2 in sporadic Creutzfeldt-Jakob disease of the VV subgroup: phenotypic and prion protein characteristics

Ignazio Cali, Gianfranco Puoti, Jason Smucny, Paul Michael Curtiss, Laura Cracco, Tetsuyuki Kitamoto, Rossana Occhipinti, Mark Lloyd Cohen, Brian Stephen Appleby, Pierluigi Gambetti*

* Address correspondence to: Pierluigi Gambetti, pxg13@case.edu

SUPPLEMENTARY MATERIALS AND METHODS

Reagents and antibodies

NaCl, Nonidet P-40, sodium deoxycholate, Tris-HCl, phosphate buffered saline (PBS), Dulbecco's PBS (D-PBS), phenylmethanesulfonyl fluoride (PMSF), proteinase K (PK), Kodak Biomax MR and XAR films were purchased from Sigma Aldrich (St. Louis, MO, USA); sodium dodecyl sulfate (SDS), β–mercaptoethanol, Tween 20, 15% Criterion Tris-HCl polyacrylamide precast gels, bromophenol blue, non-fat dry milk, Tris-buffered saline (TBS) from Bio-Rad Laboratories (Hercules, CA, USA); Odyssey blocking buffer from LI-COR Biosciences (Lincoln, NE, USA); methanol, GdnHCl solution, ethylenediaminetetraacetic acid (EDTA) from Promega (Madison, WI, USA); polyvinylidene difluoride (PVDF) membrane (Immobilon-P or Immobilon-FL) from EMD Millipore (Billerica, MA, USA); ECL and ECL plus reagents from GE Healthcare Life Sciences (Piscataway, NJ, USA). Antibodies (Abs) used were: 3F4 (to human PrP residues 106-110)[1,2], 12B2 (to human PrP residues 89-93)[3], 1E4 (to human PrP residues 97–108)[4] (Cell Sciences, Canton, MA, USA), and Tohoku-2 (To-2), which was kindly provided by Dr. Tetsuyuki Kitamoto[5]. Secondary Abs included infrared Dye (IRDye) 800CW goat anti-mouse IgG and IRDye 680RD goat anti-rabbit IgG (LI-COR Biosciences, Lincoln, NE, USA); goat anti-mouse IgG (Fc) horseradish peroxidase (HRP)-conjugate from Thermo Fisher Scientific Inc. (Waltham, MA, USA); sheep anti-mouse IgG, HRP-linked whole antibodies from GE Healthcare, Life Sciences (Piscataway, NJ, USA).

Brain homogenates

Brain homogenates (10% wt/vol) were prepared in 1X LB100 (100 mM NaCl, 0.5% Nonidet P-40, 0.5% sodium deoxycholate, 10 mM EDTA, 100 mM Tris–HCl, pH 8.0) and centrifuged at 1,000 x g for 5 min at 4 °C to collect the supernatants (S1). PK was used at 48 U/mg PK specific activity (1 U/ml is equal to ~20 µg/ml PK), while PK digestion was performed at 37 °C for 1 h. The enzymatic reaction was stopped with 3 mM final concentration of PMSF; samples were diluted with an equal volume of 2X Laemmli buffer (6% SDS, 20% glycerol, 4 mM EDTA, 5% β –mercaptoethanol, 125 mM Tris–HCl, pH 6.8) prior denaturation at 100 °C for 10 min.

Western blot analysis

To determine the prevalence of T1, T2, and T1-2, including the two T1 variants with unglycosylated isoform migrating to ~20 kDa ($T1^{20}$) and ~21 kDa ($T1^{21}$), we used 15% Tris–HCl SDS–polyacrylamide long gels (W x L: 20 cm × 20 cm; Bio-Rad PROTEAN® II xi cell system) as previously described[6,7]. Briefly, proteins were blotted onto the Immobilon-P PVDF membrane, blocked with 5% non-fat dry milk in TBS-T, and incubated with primary Abs 3F4 (1:40,000 or 1:10,000), 1E4 (1:500), 12B2 (200 ng/ml), and To-2 (1:5,000)

for 2 h. After washing with 1X TBS-T, membranes were incubated with HRP-conjugated sheep anti-mouse or donkey anti-rabbit IgG, HRP-linked whole antibodies (1:3,000). Membranes were developed by an enhanced chemiluminesce reaction using ECL and ECL plus reagents, and signal captured on MR and XAR films. Densitometric analysis of the unglycosylated resPrP^D bands visualized by chemiluminescence was performed with UN-SCAN-IT gel 5.1 software. For all the other experiments, including i) brain regional distribution of resPrP^D types, ii) PK-titration assay, and iii) conformational solubility and stability assay (CSSA), we employed the near-infrared LI-COR system as previously described[7,8]. Briefly, proteins were separated on 15% Criterion™ Tris–HCl Precast Gels (W x L: 13.3 cm × 8.7 cm), blotted onto the Immobilon-FL PVDF membrane for 2 h, blocked with the Odyssey blocking buffer and incubated with the primary Abs. Membranes were then washed with 1X DPBS-T (0.1% Tween 20 in 1X DPBS) and incubated with Abs IRDye 800CW goat anti-mouse IgG (1:15,000) or IRDye 680RD goat anti-rabbit IgG (1:15,000) for 1 h. After washing with 1X DPBS-T, membranes were developed using Odyssey infrared imaging system (LI-COR Biosciences) as described by the manufacturer. Data were analyzed with Odyssey application software V3.0.

Molecular genetics

DNA was extracted from frozen brain tissues in all cases, and genotypic analysis of PRNP coding region was performed as described previously[9,10].

Brain regional distribution of resPrP^D

The distribution of each resPrP^D type in the brain of the sCJDVV cases was determined by dividing the relative amount of resPrP^D in one brain region for the amount of resPrP^D detected in the whole brain. Each point of the profile in Figure 2 A and B is expressed as mean ± SEM.

Conformational solubility and stability assay

Conformational solubility and stability assay (CSSA) of insoluble total PrP^D (totPrP^D) and resPrP^D was performed as previously described[11]. PrP signal intensity was measured on membrane by the near-infrared LI-COR system. A dose-response equation best fitted the curves of solubility generated by CSSA, and GdnHCl$_{1/2}$ index values (expressed as mean ± SEM) were obtained with GraphPad Prism 8.1.1.

Clinical evaluation

Clinical information was gleaned from medical records. Clinical records were requested at the time of referral to the NPDPSC Autopsy Program. Clinical onset was defined as the first persistent clinical symptom consistent with a diagnosis of prion disease. Duration was defined as the time from clinical onset until death. Medical records were reviewed for the following typical symptoms helping distinguishing clinically sCJDVV1 and -VV2 such as cognitive decline (e.g, memory loss, dementia) and

cerebellar signs (e.g., ataxia, dysmetria). Brain MRI's were determined to be suggestive of prion disease as per criteria described by Zerr and coworkers[12].

Image acquisition and statistical analysis

Image acquisition was carried out with a Leica DFC 425 digital camera mounted on a Leica DM 2000 microscope. Statistical significance was determined by Student's t-test (two-tailed) in PK-titration assay, CSSA, and in experiment on brain regional distribution of resPrP^{D}. The Fisher's exact test was used when comparing clinical and pathological features among the three sCJDVV subtypes. The correlation between the percentage of T2 and the two clinical variables cognitive decline and cerebellar signs were assessed with the Pearson correlation coefficient and the linear regression. All the statistical analyses were performed using GraphPad Prism 8.1.1.

Supplementary Table S1. Neuropathological features and prevalence of sCJD subtypes with single and mixed PrP^D type.

sCJD subtypes	Histopathological phenotype	Prevalence (%)
MM(MV)1	HE: Small vacuoles affecting all layers of CC, Occ more severely than Fr CC. IHC: Diffuse or synaptic co-distributing with SD; "brush stroke-like" in Crbl.	MM1: 56[a]; 60[b]<br>MV1: 5[b]
VV1	HE: Medium-size vacuoles, ballooned neurons affecting all CC layers; astrogliosis. IHC: Faint diffuse in CC and subcortical regions; diffuse or unstained in Crbl.	2[b]; 3[c]
MM2	HE: Large, confluent vacuoles of "grape-like" type; Crbl spared. IHC: Perivacuolar and coarse in CC; Crbl Mol. L. with coarse.	4[b]; 5[a]
MV2C<br><br>MV2K	MV2C as MM2;<br><br>HE: SD with pseudo-laminar distribution; Crbl with KP; IHC: MV2K, diffuse, perineuronal, plaque-like in CC; KP and plaque-like in Crbl.	10[be]
VV2	HE: Laminar SD; atrophy of Crbl Grl. L.<br>IHC: Diffuse laminar in CC with perineuronal and plaque-like; plaque-like in Crbl.	14[b]
MM1-2	HE/IHC: Mixed -MM1 and -MM2 features related to T2:T1 ratio	43[c]; 39[a,f]
MV1-2	HE/IHC: Resembling -MM1-2 or -MV2K depending on presence/absence of KP. MV1-2 with KP rare.	23[c, f]
VV1-2	HE/IHC: Mixed -VV1 and -VV2 features related to T2:T1 ratio; VV2 features predominating in Crbl.	15[c]; 23-57[d, f]

[a] Cali *et al.*, 2009[6]; [b] Collins *et al.* 2006[13]; [c] Parchi *et al.*, 2009[14]; [d] this study; [e] includes MV2K and MV2C; [f] percentages refer to prevalence within the sCJDMM, -MV or -VV cohorts not the entire sCJD group. HE: hematoxylin-eosin; IHC: PrP immunohistochemistry; CC: cerebral cortex; Occ: occipital; Fr: frontal; SD: spongiform degeneration; Crbl: cerebellum; Mol. L.: molecular layer; Grl. L: granular layer; KP: kuru plaques; dis. dur.: disease duration; T1: disease-related PrP (PrP^D) type 1; T2: PrP^D type 2.

Supplementary Table S2. Histopathological and PrP immunohistochemical features in sCJDVV1-2 compared with sCJDVV1 and sCJDVV2.

sCJD subtype	Case #	Disease onset	Disease duration	Case #	resPrP^D T2 (%) cerebral cortex[a]	H.E. VV1-like Cerebral cortex: Medium size vacuoles[h]	H.E. VV1-like Cerebral cortex: Ballooned Neurons[i]	H.E. VV2-like Cerebral cortex: Laminar SD[j]	PrP IHC VV1-like Cerebral cortex: Widespread staining affecting all layers[k]	Case #	resPrP^D T2 (%) cerebellum	H.E. VV2-like Cerebellum (Grl. L): Atrophy score[l]	PrP IHC VV2-like Cerebellum (Grl. L): Plaque-like[l]
VV2	1	72	6	1	100	–	–	+	–	1	100	2	+
	2	56	4	2	100	–	–	+	–	2	100	2.5	+
	3	59	3	3	100	–	–	+	–	3	100	2	+
	4	72	9	4	100	–	–	+	–	4	100	3	+
	5	72	8	5	100	–	–	+	–	5	100	2.5	+
	6	64	8	6	100	–	–	+	–	6	100	2	+
	7	78	4	7	100	–	–	+	–	7	100	1	+
	8	71	4	8	100	–	–	+	–	8	100	3	+
		**68±8**[b]	**6±2**[b]		**100**	**0**[c] 0/8[d]	**0**[c] 0/8[d]	**100**[c] 8/8[d]	**0**[c] 0/8[d]		**100**	**2.2±0.6**[b]	**100**[c] 8/8[d]
VV1-2 vs.VV2[e]						<0.03	NS	NS	<0.04			<0.03[f]	NS
VV1-2	1	55	4	2	100	–	–	+	–	1	100	NA	+
	2	70	3	6	100	–	–	+	–	3	100	1.5	+
	3	70	17	1	97	–	–	+	–	4	100	3	+
	4	66	4	3	94	–	–	+	+	5	100	2.25	+
	5	74	5	4	93	–	–	+	–	6	100	2.25	+
	6	73	7	5	90	–	–	+	–	8	100	2	+
	7	44	7	7	88	–	–	+	–	9	100	0.5	+
	8	45	5	8	87	–	–	+	–	10	100	1.5	+
	9	57	8	9	69	+	+	–	+	11	100	1.5	+

	10	60	5	10	63	+	−	+	−	12	100	NA	NA
	11	79	8	11	48	+	−	−	+	14	100	1	+
	12	84	4	12	45	+	−	+	−	7	99	1	+
	13	60	5	13	38	+	−	−	+	2	76	2.25	+
	14	68	4	14	26	−	+	+	−	13	64	2.25	+
	15	69	26	17	9	+	+	−	+	16	10	0	−
	16	71	18	16	6	+	+	−	+	15	0	1	+[g]
	17	66	13	18	4	+	+	−	+	17	ND	1	−
	18	52	7	15	2	+	−	−	+	18	ND	0.5	−
		**65±11**[b]	**8±6**[b]		**59±37**[b]	**50**[c]	**28**[c]	**61**[c]	**44**[c]		**84±33**[b]	**1.5±0.8**[b]	**82**[c]
						9/18[d]	5/18[d]	11/18[d]	8/18[d]				14/17[d]
VV1	1	34	14	1	0	+	+	−	+	1	0	0.5	−
	2	30	10	2	0	+	+	−	+	2	0	0.5	−
	3	34	13	3	0	+	+	−	+	3	0	0.5	−
	4	25	8	4	0	NA	+	NA	+	4	0	1.5	−
	5	38	12	5	0	+	+	−	+	5	0	1.5	−
		32±5[b]	11±2[b]		**0**	**100**[c]	**100**[c]	**0**[c]	**100**[c]		**0**	**0.9±0.5**[b]	**0**[c]
						4/4[d]	5/5[d]	0/4[d]	5/5[d]				0/5[d]
VV1-2 vs.VV1[e]						NS	<0.008	NS	<0.05			NS[f]	<0.003

[a] See Table 1 for technical details; [b,c] Expressed as [b] mean ± standard deviation and [c] percentage; [d] cases with the feature listed/total cases examined; [e] Fisher's exact test; [f] Student's t-test. [g] Scattered foci of plaque-like PrP immunostaining; NS: not significant; H.E.: hematoxylin-eosin; IHC: immunohistochemistry. [h,i, k] These three cerebral cortex (CC) pathologies inversely correlated (r= - 0.96-0.99) with T2 relative amount (%T2). [j] Laminar spongiform degeneration (SD) directly correlated (r= 0.99) with %T2 of CC in -VV1-2 cases 1-8, 9-14 and 15-18. [l] Plaque-like PrP and atrophy positively correlated with %T2 of cerebellum (crbl) (r= 0.99-0.93). Prevalence of individual pathological features in CC and crbl of -VV1-2 >50% T2 and -VV1-2 <50% T2 did not differ from those of -VV2 and -VV1, respectively. Pearson's test was used to assess correlations.

SUPPLEMENTARY FIGURE LEGENDS

Supplementary Figure S1. **PK-titration assay to discriminate between *bona fide* resPrP^D T1 and partially cleaved fragments**. Supernatant (S1) was digested with increasing concentration of PK and probed with the Ab 12B2 (See Materials and Methods for more details). **A**: $PK_{1/2}$ in -VV2 was on average about 17-fold smaller than the $PK_{1/2}$ index in -VV1 (0.4 vs. 6.9 U/ml; P<0.004) defining the 12B2-immunoreactive PrP^D as partially cleaved fragments. Compared to the resPrP^D amount detected at 0.6 U/ml PK (i.e. 100%), only 8% resPrP^D in -VV1, but ~ 70% in -VV2, was digested following incubation with 5 U/ml PK (P<0.002). Each curve (not shown) of $PK_{1/2}$ determination was fitted by a one phase decay equation. **B**: Representative WB of -VV1 (top) and -VV2 (bottom) following digestion with increasing PK concentrations. Partially cleaved fragments in -VV2 are visible up to ~ 2.5-5 U/ml, *bona fide* resPrP^D T1 up to ~ 40-80 U/ml in -VV1. **C**: No resPrP^D was detected by type 1 specific Ab 12B2 from any of the brain regions examined in 8 cases of -VV2 (one representative case shown here) after treatment with 10 U/ml PK.

Supplementary Figure S2. **Uncropped Western blots of Figure 1B**. Red numbers atop of each WB refer to resPrP^D showed in lanes 1-11.

Supplementary Figure S1

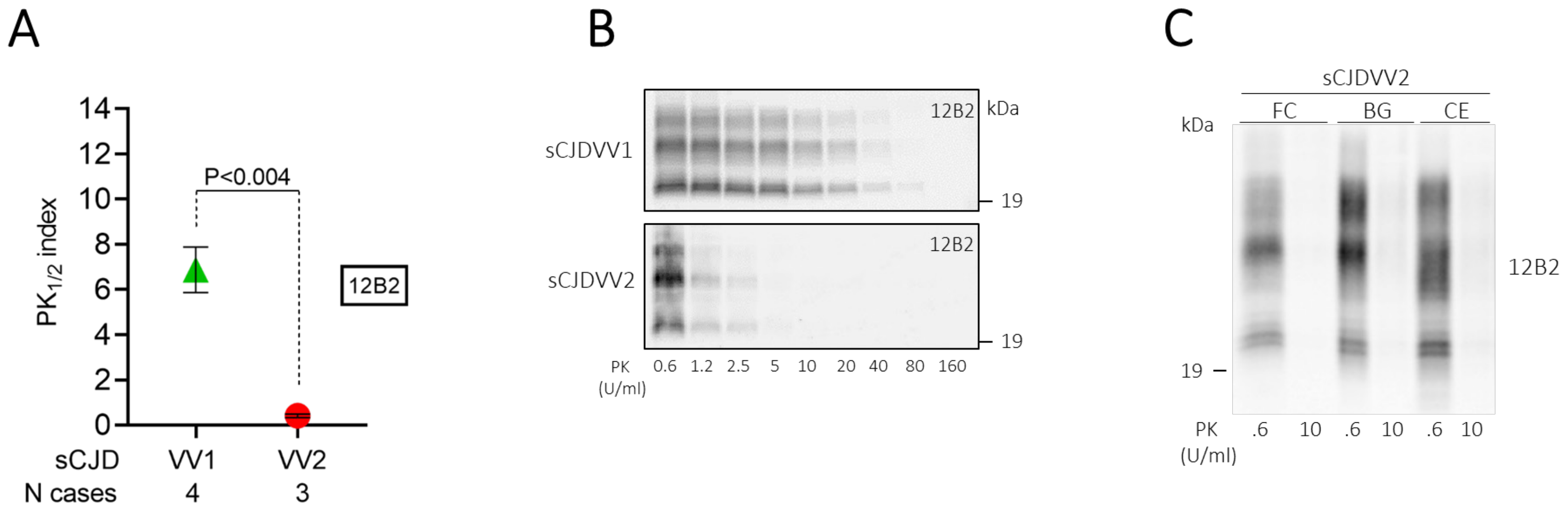

Supplementary Figure S2

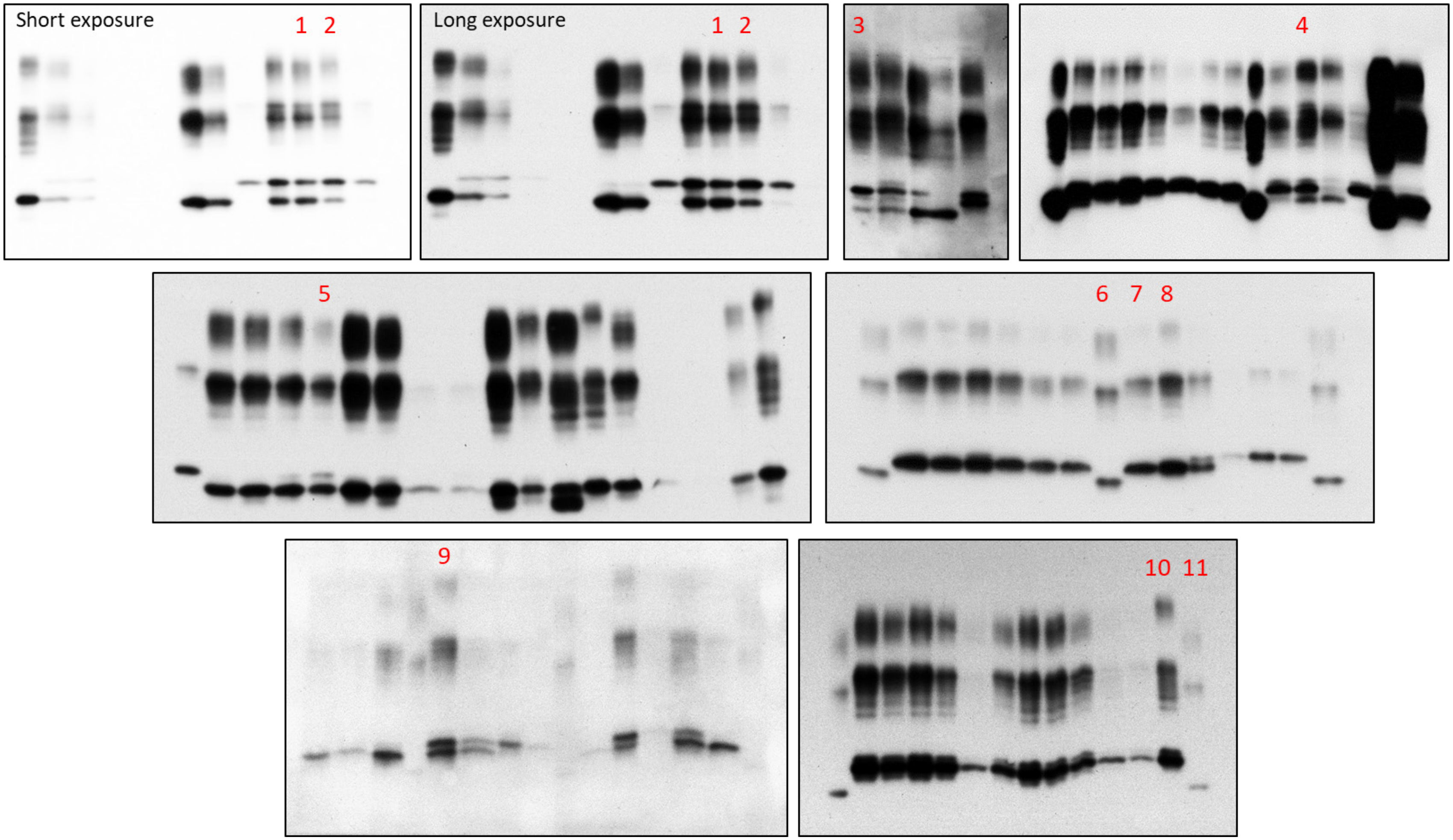

References

1. Kascsak, R. J. *et al.* Mouse polyclonal and monoclonal antibody to scrapie-associated fibril proteins. *J. Virol.* **61**, 3688–3693 (1987).
2. Zou, W.-Q. *et al.* PrP conformational transitions alter species preference of a PrP-specific antibody. *J. Biol. Chem.* **285**, 13874–13884, DOI: 10.1074/jbc.M109.088831 (2010).
3. Langeveld, J. P. M. *et al.* Rapid and discriminatory diagnosis of scrapie and BSE in retro-pharyngeal lymph nodes of sheep. *BMC Vet. Res.* **2**, 19, DOI: 10.1186/1746-6148-2-19 (2006).
4. Yuan, J. *et al.* Accessibility of a critical prion protein region involved in strain recognition and its implications for the early detection of prions. *Cell. Mol. Life Sci. CMLS* **65**, 631–643, DOI: 10.1007/s00018-007-7478-z (2008).
5. Kobayashi, A. *et al.* Experimental verification of a traceback phenomenon in prion infection. *J. Virol.* **84**, 3230–3238, DOI: 10.1128/JVI.02387-09 (2010).
6. Cali, I. *et al.* Co-existence of scrapie prion protein types 1 and 2 in sporadic Creutzfeldt-Jakob disease: its effect on the phenotype and prion-type characteristics. *Brain J. Neurol.* **132**, 2643–2658, DOI: 10.1093/brain/awp196 (2009).
7. Cali, I. *et al.* Iatrogenic Creutzfeldt-Jakob disease with Amyloid-β pathology: an international study. *Acta Neuropathol. Commun.* **6**, 5, DOI: 10.1186/s40478-017-0503-z (2018).
8. Cali, I. *et al.* Distinct pathological phenotypes of Creutzfeldt-Jakob disease in recipients of prion-contaminated growth hormone. *Acta Neuropathol. Commun.* **3**, 37, DOI: 10.1186/s40478-015-0214-2 (2015).
9. Parchi, P. *et al.* Molecular basis of phenotypic variability in sporadic Creutzfeldt-Jakob disease. *Ann. Neurol.* **39**, 767–778, DOI: 10.1002/ana.410390613 (1996).
10. Parchi, P. *et al.* Genetic influence on the structural variations of the abnormal prion protein. *Proc. Natl. Acad. Sci. U. S. A.* **97**, 10168–10172, DOI: 10.1073/pnas.97.18.10168 (2000).
11. Cracco, L. *et al.* Novel strain properties distinguishing sporadic prion diseases sharing prion protein genotype and prion type. *Sci. Rep.* **7**, 38280, DOI: 10.1038/srep38280 (2017).
12. Zerr, I. *et al.* Updated clinical diagnostic criteria for sporadic Creutzfeldt-Jakob disease. *Brain J. Neurol.* **132**, 2659–2668, DOI: 10.1093/brain/awp191 (2009).
13. Collins, S. J. *et al.* Determinants of diagnostic investigation sensitivities across the clinical spectrum of sporadic Creutzfeldt-Jakob disease. *Brain J. Neurol.* **129**, 2278–2287, DOI: 10.1093/brain/awl159 (2006).
14. Parchi, P. *et al.* Incidence and spectrum of sporadic Creutzfeldt-Jakob disease variants with mixed phenotype and co-occurrence of PrPSc types: an updated classification. *Acta Neuropathol. (Berl.)* **118**, 659–671, DOI: 10.1007/s00401-009-0585-1 (2009).
